# Superantigens as Contributors to the Etiology of Kawasaki Disease Shock Syndrome

**DOI:** 10.3390/jcm15187268

**Published:** 2026-09-18

**Authors:** Marco A. Yamazaki-Nakashimada, Mónica A. Montoya-Guzmán, Giselle Quezada-Ortega, Maria Elisa Drago-Serrano, Andrea Iglesias-Amaya, Adrian Rosales-Hernández, Chiharu Murata, Camilo Rodríguez-López, Marycarmen Godínez-Victoria

**Affiliations:** 1 Immunology Department, Instituto Nacional de Pediatría, Mexico City 04530, Mexico; yzki71@yahoo.com.mx (M.A.Y.-N.); andrea.iglesias18@gmail.com (A.I.-A.); telurio17@gmail.com (A.R.-H.); 2 Sección de Estudios de Posgrado e Investigación, Escuela Superior de Medicina, Instituto Politécnico Nacional, Mexico City 11340, Mexico; monguz22@hotmail.com (M.A.M.-G.); qogiselle@outlook.com (G.Q.-O.); 3 Departamento de Sistemas Biológicos, Universidad Autónoma Metropolitana, Unidad Xochimilco, Mexico City 04960, Mexico; mdrago@correo.xoc.uam.mx; 4 Research Methodology Department, National Institute of Pediatrics, Health Secretariat, Mexico City 04530, Mexico; chiharumurata@gmail.com; 5 Laboratorio Clínico del Hospital General de Zona No. 57, Instituto Mexicano del Seguro Social, Estado de Mexico 54769, Mexico; camilorodriguez.qfb@gmail.com

**Keywords:** Kawasaki diseases shock syndrome, Kawasaki, disease, superantigen, staphylococcus enterotoxin A, T cell receptor Vβ2

## Abstract

**Background**: Before the COVID-19 pandemic, the higher prevalence of gastrointestinal symptoms, severe inflammatory response, and cardiac failure in patients with Kawasaki Disease Shock Syndrome (KDSS) lets us hypothesize that KDSS is triggered by superantigens produced by bacteria that colonize the gastrointestinal tract and activate TCRVβ2+ and TCRVβ8+ cells, leading to a massive proinflammatory stage and cardiovascular instability. **Methods**: This is a case–control study in patients with KDSS (cases, 8 patients) or with Kawasaki disease (KD; controls, 71 patients), in the acute phase before intravenous immunoglobulin (IVIG) treatment, from the National Institute of Pediatrics, Mexico City, from 2016 to 2019. Clinical and laboratory data were obtained with quantification of TCRVβ2+ and TCRVβ8+ T cells by flow cytometry. Superantigen-associated gene-positive bacterial isolates from stool cultures were obtained by final point-PCR assay. Statistical analysis was done with one-way ANOVA and Welch’s post hoc test, Chi-square test and odds ratio (OR). Statistical significance was defined as *p* < 0.05. **Results**: The peripheral proportion of CD4+TCRVβ2+ T cells was lower in the KDSS group than in the KD group, whereas the CD25-positive T-cell subsets showed no statistically significant differences between groups. Significant associations between staphylococcus enterotoxin A (SEA)/KDSS [OR = 23.3, CI95% = 1.8–296.2, *p* = 0.014] and superantigen/coronary aneurysms (*p* < 0.001) were found. **Conclusions**: These results suggest that SEA produced by bacteria that colonize the gastrointestinal tract and cross the intestinal barrier may activate CD3+/CD4+/TCRVβ2+ T cells, as a trigger of shock in a group of patients with KD. Also, each subtype of KD could have different etiologies explaining the inconsistencies in the detection of superantigens in patients with KD.

## 1. Introduction

Kawasaki disease (KD) is an acute, self-limited systemic vasculitis that predominantly affects small- and medium-sized arteries, with a particular predilection for the coronary arteries. It is the leading cause of acquired heart disease in children in developed countries. Early diagnosis and prompt treatment with intravenous immunoglobulin (IVIG) significantly reduce the risk of coronary artery abnormalities, which remain the most serious complication of the disease [1,2].

The diagnosis of KD is primarily clinical. According to current international guidelines, complete (classic) KD is defined by persistent fever accompanied by at least four of the five principal clinical features: bilateral non-exudative conjunctival injection, oral mucosal changes, polymorphous rash, changes in the extremities, and cervical lymphadenopathy [1,2]. Incomplete KD refers to patients with prolonged fever and only two or three of the principal clinical features, in whom the diagnosis is supported by elevated inflammatory markers, compatible laboratory findings, and echocardiographic evidence of coronary involvement [1,2]. This presentation is particularly common in infants younger than six months and older children, populations at increased risk of delayed diagnosis and coronary complications [1,2].

Importantly, incomplete KD should not be considered synonymous with atypical KD. Current guidelines reserve the term atypical KD for patients presenting with uncommon manifestations, including neurological, gastrointestinal, renal, pulmonary, hepatobiliary, or musculoskeletal involvement that falls outside the classical diagnostic criteria [1,2]. Distinguishing between incomplete and atypical KD has important clinical implications because delayed recognition of these presentations is associated with an increased likelihood of coronary artery aneurysm formation and other cardiovascular sequelae [1,3].

The clinical spectrum of this disease is wide, with severe forms presenting with shock (Kawasaki disease shock syndrome, KDSS) or macrophage activation syndrome (MAS) [4,5,6]. KDSS is an uncommon presentation of KD and has been associated with more severe markers of inflammation, cardiac failure, and abdominal symptoms [5,7]. Unlike the Japanese population, the Hispanic population has a higher incidence of coronary aneurysms and KDSS [8].

The etiology of KD remains uncertain; an infectious trigger is now accepted as the genesis of an immune response in genetically predisposed children. In 1991, Furukawa et al. proposed that KD could be caused by superantigens (SAgs) [9]. Initial studies suggested involvement of SAgs in the development of KD, but later, other studies showed inconsistent and inconclusive results. The aim of this study performed before the COVID-19 pandemic was to explore the hypothesis that KDSS is triggered by SAgs produced by bacteria that colonize the gastrointestinal tract and activate TCRVβ2+ and TCRVβ8+ T cells, leading to a massive proinflammatory stage and cardiovascular instability.

## 2. Materials and Methods

### 2.1. Study Subjects

This is a case–control, epidemiological, analytical, non-experimental, and prospective study, carried out from 8 November 2016 to 30 June 2019, at the National Institute of Pediatrics in Mexico City. Written informed consent was obtained from the parents or guardians of all participants before enrolment. Study subjects were selected based on the presence (cases) or absence of shock syndrome (controls) in patients diagnosed with KD in the acute phase prior to treatment with intravenous gamma globulin (IVIG). The diagnosis of KD and aneurysm was established with the criteria of the American Heart Association (AHA) [2]. KDSS was defined as KD associated with hemodynamic instability characterized by hypotension or clinical manifestations of poor peripheral perfusion requiring fluid resuscitation and/or vasoactive support. Hypotension was defined according to age-specific systolic blood pressure criteria or as a decrease of ≥20% in systolic blood pressure from the patient’s baseline. The diagnosis of KDSS did not require the presence of ventricular dysfunction, as shock in KD may result from vasodilation, capillary leak, myocardial dysfunction, or a combination of these mechanisms. All diagnoses were established by the treating clinical team based on the clinical, laboratory, and echocardiographic findings available during hospitalization.

Coronary artery involvement was assessed by two-dimensional transthoracic echocardiography during the acute phase of Kawasaki disease and during subsequent clinical follow-up. Echocardiographic examinations were not performed under blinded conditions because the study was based on clinical echocardiographic evaluation during patient care. Internal luminal diameters of the left main coronary artery, left anterior descending coronary artery, and right coronary artery were measured and normalized for body surface area using Z scores according to the 2017 American Heart Association (AHA) recommendations [2]. Coronary artery dilation was defined as a Z score ≥ 2.0 and <2.5. Coronary artery aneurysms were classified according to AHA criteria as small (Z score ≥ 2.5 and <5.0), medium (Z score ≥ 5.0 and <10.0), and large or giant (Z score ≥10.0). The maximum coronary artery Z score observed during the illness was used to classify the severity of coronary involvement.

During follow-up, patients were monitored according to Kawasaki’s institutional disease management protocol, including clinical evaluation and cardiovascular assessment as clinically indicated. The follow-up period of patients was individualized according to cardiovascular condition and systemic involvement, particularly in patients with KDSS and atypical KD.

### 2.2. Collection and Storage of Samples

On arrival, a peripheral venous blood sample (2 mL) was taken and collected in a tube with EDTA-Na2 to prevent coagulation and was stored under refrigeration (4–8 °C) for a maximum period of 24 h until its analysis by flow cytometry. In addition, a stool sample (2 cm^2^) was collected for microbiological cultures and identification of SAg-associated gene-positive bacterial isolates; the sample was kept under refrigeration (4–8 °C) for a maximum period of 72 h until processing.

### 2.3. Frequency Determination of Lymphoid Subpopulations

The determination of percentages of CD3+, CD3+/CD4+, CD3+/CD8+, CD3+/CD4+/TCRVβ2+, CD3+/CD8+/TCRVβ2+, CD3+/CD4+/TCRVβ8+ and CD3+/CD4+/TCRVβ8+, as well as CD25 expression, considered as an activation marker of lymphocytes, was assayed by flow cytometry. Cell staining was performed using the following anti-human monoclonal antibodies from BD Pharmigen (San Jose, CA, USA): PE-Cy7-CD8 (catalog 557746), PerCP-CD4 (catalog 550631), APC-H7-CD3 (catalog 560176), APC-CD25 (catalog 555434), and FITC-Vβ8 (catalog 555606), as well as PE-Vβ2 from Beckman Coulter (Brea, CA, USA) (catalog 4116015). Briefly, 100 μL of blood was transferred to each tube and erythrocytes were lysed using 2 mL of 1X lysis buffer according to the manufacturer’s instructions (1–2 Step Fix/Lyse Solution 10X, Cat. 00-5333-54, Invitrogen, Carlsbad, CA, USA) and washed using IX phosphate buffer saline (PBS); for this, samples were centrifuged at 1500 rpm for 5 min and the supernatant was discarded. After this, samples were incubated with a mix of all antibodies in darkness at room temperature for 30 min and finally washed and resuspended in 4% paraformaldehyde in PBS solution for analysis in an BD FACSAria^TM^ Fusion cell sorter 656700 (Becton Dickinson and Company, BD Biosciences, San Jose, CA, USA). Twenty thousand events were acquired from the lymphocyte region in the FSS/SCC dot-plot. The acquisition and analysis of the samples was carried out with BD FACSDiva software version 8.0.1. Data are reported as percentages of positive cells.

### 2.4. Bacterial Culture and Detection of SAg-Associated Genes

For microbiological culture, a suspension with 1 g of the stool sample was made in 20 mL of sterile PBS and selective culture media were inoculated. The culture media used were EMB Agar (BD Bioxon catalog 210600, Franklin Lakes, NJ, USA), MacConkey Agar (MCD LAB catalog 7112, Daejeon, Republic of Korea), Salt mannitol agar (MCD LAB catalog 7152), Blood Agar and Chocolate Agar (MARCK catalog 10886, Darmstadt, Germany). These culture media were selected to facilitate the recovery of different bacterial groups of interest, including bacterial groups in which the superantigens investigated in this study have been described.

Initially, total bacterial DNA extracted directly from stool samples using the PureLink™ Genomic DNA Mini Kit (catalog K182002, Invitrogen, Carlsbad, CA USA) was subjected to endpoint PCR. However, none of the target genes were amplified from the DNA obtained directly from stool. Therefore, bacterial DNA extracted from the stool-derived cultured isolates was subsequently used for individual endpoint PCR assays targeting the genes encoding staphylococcal enterotoxins *A*, *B*, and *C* (*sea*, *seb*, *and sec*), streptococcal pyrogenic exotoxins A, B, and C (*speA*, *speB*, and *speC*), and toxic shock syndrome toxin-1 (*tst-1*). The reactions were carried out at a volume of 25 μL using Taq PCR Core Kit (1000 U, catalog 201225, Qiagen, Brand, Germany), according to the manufacturer’s instructions. The primers used for each gene are shown in Table 1.

The StepOne thermal cycler from Applied Biosystem (Foster City, CA, USA) was used under the following thermal parameters: activation at 94 °C for 5 min; denaturation at 94 °C for 1 min; alignment at 55 °C for 1 min; elongation at 72 °C for 1 min; repetition for 29 cycles; termination at 72 °C for 10 min; cooling at 4 °C; and pause.

PCR products were analyzed by electrophoresis with 1.5% agarose gels, stained with 1 μL of GelRed (Biotium catalog 41003, Fremont, CA, USA). As a molecular weight marker, 100 pb DNA Ladder RTU was used (catalog DM001-R500, brand Gene Direx, Taoyuan, Taiwan).

Once the gel was placed inside the electrophoresis chamber, it was run at 100 volts, 400 mA and 100 W for 40 min or until the dye migrated up to three-quarters of the gel. The visualization was made by means of gel documentation system Fusion SL (Viber Laurmat, Marne La Vallee, Francia) by the incidence of ultraviolet light of 300 nm wavelength, allowing the fluorescence of the red gel, which is incorporated into the nucleic acid during the development of the gel. Finally, to record the results, a photograph of the gel was taken with the DNA bands, and those that were of the expected size were considered positive for SAg according to the reaction.

The PCR assay was used to detect the presence of SAg-associated genes in cultured bacterial isolates and was not intended to demonstrate toxin expression, secretion, or biological activity. Therefore, PCR-positive isolates were considered to carry the corresponding SAg-associated gene, without assuming that the encoded toxin was actively produced.

### 2.5. Bacterial Identification

Bacterial identification and susceptibility were assessed in Microscan Autoscan-4 System Beckman Coulter^®^ (B1018-280—Beckman Coulter, Brea, CA, USA).

### 2.6. Statistical Analysis

Data obtained from patients were structured in a database for case–control comparison. Differences in continuous variables (means) between cases and controls were evaluated using Student’s *t*-test. Homoscedasticity was assessed using Levene’s test, and Welch’s *t*-test was applied in instances of unequal variances. Binary categorical comparisons were assessed using two-sided Fisher exact tests. For the *sea*–KDSS comparison, the unadjusted sample odds ratio was calculated from the 2 × 2 table; its approximate 95% confidence interval was calculated on the log-odds-ratio scale using the Wald method. A targeted sensitivity analysis excluded each *sea*-positive KDSS patient in turn and repeated Fisher’s exact test. Missing *sea* results were excluded from this comparison and were not treated as negative. The revised *sea*–KDSS analysis is unadjusted. A multivariable model was not retained because the small number of KDSS events and sparse exposure data did not support a sufficiently reliable, adjusted analysis. Statistical significance was set at *p* < 0.05. All statistical analyses were performed using JMP^®^ Pro version 13.0.0.

## 3. Results

### 3.1. Characteristics of the Population

The cohort comprised 91 patients, including eight with KDSS and 83 with KD without shock. *sea* results were available for 79 patients (eight and 71, respectively). Twelve patients with KD had no evaluable *sea* result because stool specimens had not been obtained. Missing results were not classified as negative. Denominators for other measurements were determined separately. Of the total number of patients (91), 63 (69%) were male and 28 (31%) were females, with no significant difference observed with respect to the distribution by sex between cases [female patients, 2/8 (25%)] and controls [female patients, 26/83 (31%); *p* = 0.711]. Regarding the age of presentation, no significant differences were observed between cases [median 33 months (p25, p75 = 9, 54)] and controls [median 27 months (p25, p75 = 15, 55); *p* = 0.924]. This was also the case for the type of origin (urban or rural), observing an urban origin in 6/8 (75%) patients in the KDSS group and 64/83 (77%) patients in the KD group (*p* = 0.893). Twelve patients from the KD group were eliminated from the analysis of association of variables due to a lack of stool or blood samples but were considered for the report on the clinical manifestations of KDSS and KD in the Mexican population.

### 3.2. Clinical Manifestations

Incomplete KD occurred in 28/91 (30.8%) of the total study population, with no significant difference observed between the case and control groups [2/8 (25%) in the KDSS group and 26/83 (31%) in the KD group, *p* = 0.582].

In relation to the atypical form of KD, no seizures, aseptic meningitis, or pneumonitis were reported. A change in consciousness was observed in one patient from the KDSS group (12.5%). Otitis (1 patient, 1.2%), sterile pyuria (1 patient, 1.2%), and adenopathy (43 patients, 51.8%) only occurred in the KD group, but not in the KDSS group.

Table 2 shows the clinical manifestations presented by patients at the time of diagnosis, with the presence of abdominal pain being more frequent in the KDSS group (*p* = 0.005 vs. KD group) and “strawberry” tongue being more frequent in the KD group (*p* = 0.010 vs. KDSS group). The rest of the clinical manifestations showed no significant differences between the two groups. Among gastrointestinal manifestations (vomiting, abdominal pain, and diarrhea), it was observed that 100% of patients in the KDSS group presented at least one of these manifestations compared to 43% of the KD group.

### 3.3. T Cell Subpopulations

**Figure 1:**
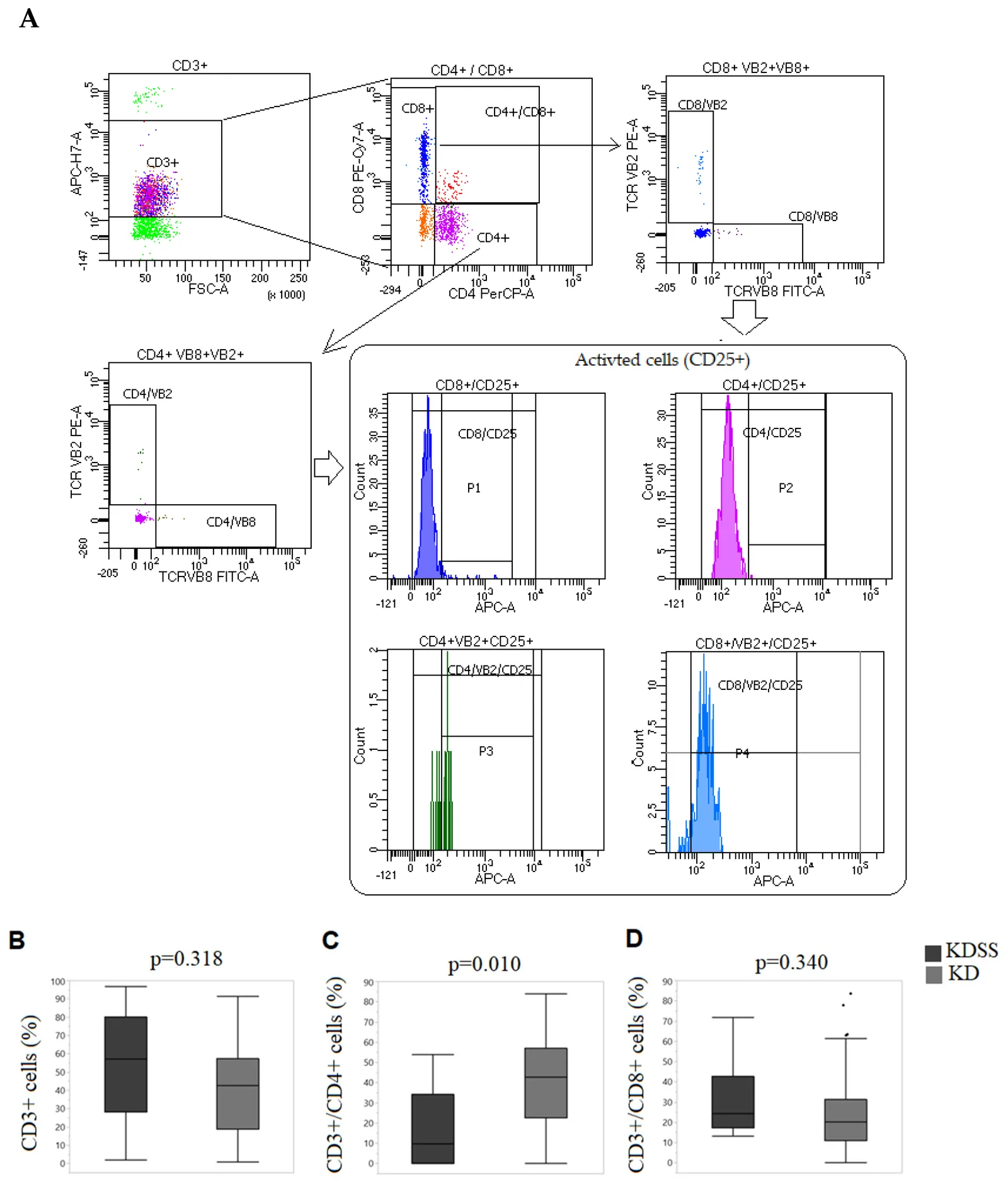
Percentage of T cell subpopulations in patients with Kawasaki disease-associated shock syndrome (KDSS group) compared to Kawasaki disease with normal hemodynamic condition (KD group). (**A**) Flow cytometry gating strategy for the identification of activated T-cell subsets. Representative dot plots illustrating the sequential gating strategy used to identify T-cell populations. First, CD3+ cells were selected. CD3+ cells were subsequently separated into CD4+ and CD8+ T-cell populations based on CD4 and CD8 expression. Within the CD8+ or CD4+ populations, cells expressing T-cell receptor Vβ2 and Vβ8 were identified. Subsequently, CD25+ cells were identified within the CD8+ and CD4+ T-cell populations, and the corresponding Vβ2+ subsets were further characterized. The final plots show the CD8+/Vβ2+/CD25+ and CD4+/Vβ2+/CD25+ populations (P3 and P4, respectively), as well as the CD8+/CD25+ and CD4+/CD25+ populations (P1 and P2). Arrows indicate the sequential gating strategy. (**B**) Total T cells (CD3+ cells); (**C**) CD3+/CD4+ T cells; (**D**) CD3+/CD8+ T cells. Horizontal lines: midline of the box—median, the bottom line of the box—1st quartile (Q1, 25th), the top line of the box—3rd quartile (Q3, 75th), and whiskers—minimum and maximum observations.

The percentage of total lymphocytes showed no significant difference between the groups (Figure 1A). The percentage of CD4+ and CD8+ T cells showed a significant decrease in the KDSS group compared to controls (*p* = 0.010, Figure 1B), and no significant difference was observed for CD8+ T cells between both groups (Figure 1C).

**Figure 2:**
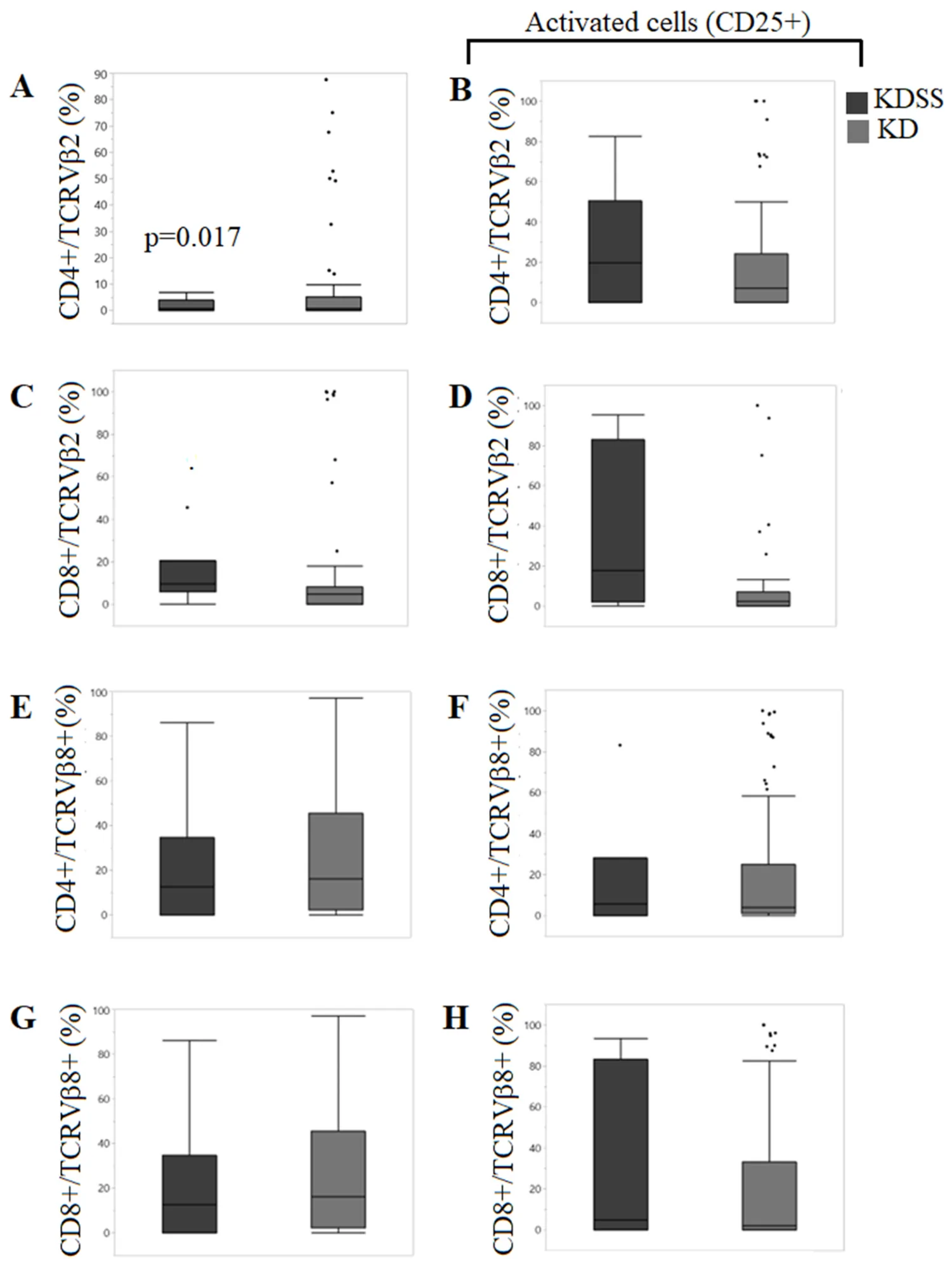
Percentage of T cell subpopulations (CD3+/CD4+ or CD3+/CD8+ cells) bearing TCR Vβ2 or Vβ8 in patients with Kawasaki disease-associated shock syndrome (KDSS group) compared to Kawasaki disease with normal hemodynamic condition (KD group). Total percentages of the T cell subpopulations (CD3+) are shown in left column: (**A**) CD4+/TCRVβ2+ T cells; (**C**) CD8+/TCRVβ2+ T cells; (**E**) CD4+/TCRVβ8+ T cells; (**G**) CD8+/TCRVβ8+ T cells. Percentages of the activated T cell subpopulations, identified by CD25+ expression, are shown in right column: (**B**) CD4+/TCRVβ2+/CD25+ T cells; (**D**) CD8+/TCRVβ2+/CD25+ T cells; (**F**) CD4+/TCRVβ8+/CD25+ T cells; (**H**) CD8+/TCRVβ8+/CD25+ T cells. Horizontal lines: midline of the box—median, the bottom line of the box—1st quartile (Q1, 25th), the top line of the box—3rd quartile (Q3, 75th), and whiskers—minimum and maximum observations.

Regarding the percentage of CD4+ and CD8+ T cells bearing TCRVβ2, a significant decrease in the percentage of CD4+TCRVβ2 T cells was observed in the KDSS group compared to the KD group (*p* = 0.017, Figure 2A); however, the percentage of CD8+TCRVβ2+ T cells did not show significant differences between both groups (*p* = 0.985, Figure 2C). Regarding the percentage of CD4+/TCRVβ8+ T cells (Figure 2E) and CD8+/TCRVβ8+ T cells (Figure 2G), no significant differences were observed between the KDSS and KD groups.

The CD25-positive CD4+Vβ2+ (Figure 2B), CD8+Vβ2+ (Figure 2D), CD4+Vβ8+ (Figure 2F), and CD8+Vβ8+ (Figure 2H) subsets showed no statistically significant differences between groups. However, the KDSS group had a trend toward a higher percentage of activated CD4+Vβ2+ T cells (Figure 2B) and CD8+Vβ2+ T cells (Figure 2D) versus the KD group (Figure 2).

### 3.4. Superantigen Staphylococcus Enterotoxin A (SEA) Was More Frequent in KDSS

The superantigens *sea*, *seb*, *sec*, *tsst-1*, *speA*, *speB* and *speC* were detected in bacterial DNA isolated from the stool cultures of patients with KDSS and KD by the endpoint PCR technique (Table 3). The results showed that *sea* was detected in 2/8 KDSS patients (25.0%) and 1/71 patients with KD without shock (1.4%). The unadjusted sample OR was 23.33 (approximate Wald 95% CI 1.84–296.19; two-sided Fisher exact *p* = 0.02585). The frequency of positive stool samples for the rest of the SAgs showed no significant differences between the groups. Excluding either SEA-positive KDSS patient changed the comparison to 1/7 versus 1/71 (*p* = 0.17249).

### 3.5. Cardiovascular Manifestations and Superantigens

In total, 50.6% of patients presented some type of cardiac manifestation and 17.7% presented coronary injury. The cardiovascular manifestations observed were pericardial effusion (34.2%), myocarditis (8.9%), pericarditis (29.1%), coronary aneurysms (8.9%), coronary ectasia (13.9%) and valvular insufficiency (16.5%). Statistical analysis between patients with or without SAgs showed no significant differences in the frequency of cardiovascular manifestations.

The presence of any SAg had a positive association with the development of coronary aneurysms (*p* < 0.001). However, no significant statistical association was observed between each of the SAgs.

### 3.6. Bacteria That Colonize the Gastrointestinal Tract

Table 4 shows the bacteria isolated from the stools of patients with KDSS and KD prior to IVIG treatment. The most frequently isolated bacteria were *Enterobacter cloacae*, *Escherichia coli*, *Staphylococcus haemolyticus*, *Staphylococcus aureus* and *Streptococcus pyogenes.* Significant differences between cases and controls were not observed. High antimicrobial resistance against amoxicillin/clavulanic acid, amoxicillin/sulbactam, ceftriaxone, clindamycin, oxacillin, and penicillin was observed in more than 80% of isolated bacteria; *S. haemoliticus* and *S. sciuri* had greater antibiotic resistance.

## 4. Discussion

KD is a disease of unknown etiology. It has been suggested that KD is triggered by the presence of SAg-producing bacteria, present in the respirator-y tract or gastrointestinal tract, that activate circulating or intestinal resident TCR Vβ2+. Elevated levels of circulating Vβ2+ and Vβ8.1+ T cells compared to the KD group were observed in patients with complete KD in the acute phase and only Vβ2 T cells in the lamina propria from small intestinal mucosa [10]. Vβ-5, -8, -12 and -19-expressing T cells were also increased in patients with KD compared to healthy controls [11]. The mRNA expression of Vβ9 and Vβ15 was decreased in the acute phase of KD [12]. Other Vβ subsets (Vβ-5.1, -5.2, -6.7, and -12) in circulating T cells [12,13] or Vβ-5a, -5b, -5c, -6a, -8a, and -12a in T cells from the small intestinal mucosa [10] were not increased in the acute phase of KD. Recent studies on the selective use of Vβ2 supported the hypothesis that an SAg is involved in the pathogenesis of KD [10,11,14] and that activation of TCR Vβ2+ T cells during the acute phase is caused by SAgs produced by bacteria colonizing the small intestinal mucosa [10] or throat [11] of these patients [15]. In other studies, bacteria producing toxins, such as toxic shock syndrome toxin (TSST)-secreting *Staphylococcus aureus* [16,17] and streptococcal pyrogenic exotoxin (SPE)-B and -C, were isolated from KD patients, suggesting that these SAgs could activate Vβ2+, Vβ8.1+ T cells [16,18] and Vβ6.5+ T cells [14]. However, contradictory results were observed in other studies, where no expansion of any Vbeta (Vβ) family (CD4+ T cells bearing TCR Vβ2, Vβ5.1, Vβ6.7, Vβ8, Vβ12.1, and Vβ19) was present in the acute phase. Recent data before the COVID-19 pandemic suggested that one or more conventional antigens drive the T cell immune response in KD and argue against a role for SAgs in the disease process [19,20,21]. In addition, the lack of serum antibodies for TSST-1, SEA, SEB, SEC, SSA, and MF in the acute phase did not support the involvement of toxin-producing staphylococci in KD [22,23].

On the other hand, Vβ2 expansion in both the CD4+ and CD8+ T cell subsets supports the concept that the activation of infiltrating Vβ2+ T cells and presence of staphylococcal exfoliative toxin are involved in the cardiovascular damage associated with KS [24,25,26].

Patients with KDSS have in common a strong inflammatory response with the elevation of acute-phase reactants, severe cardiovascular compromise, and the presence of gastrointestinal manifestations, observed in 91% of patients compared to the rest of the patients with KD (30%). The aim of our study was to evaluate the hypothesis that KDSS is triggered by SAgs produced by enterobacteria colonizing the gastrointestinal tract. We investigated the association of the diagnosis of KDSS or KD with the activation of TCRVβ2+ and TCRVβ8+ T cells and the presence of SAg-associated gene-positive bacterial isolates in the gastrointestinal tract.

In this study, we identified SAg-associated gene-positive bacterial isolates that colonize the gastrointestinal tract and evaluated whether the presence of these superantigens activates TCRVβ2+ and Vβ8+ T cells in the peripherical blood of patients with KDSS, favoring a proinflammatory state that allows the development of shock syndrome. All results were compared to patients with the incomplete and complete forms of KD, which were considered as the control group. We evaluated the percentage of CD4+ and CD8+ T cells bearing TCRVβ2 or Vβ8. Published pediatric data provide contextual Vβ2/Vβ8 frequencies [27], but differences in laboratory methods and population definitions limit direct comparison. The present study compares KDSS with KD without shock. Without a concurrently assessed healthy group, it cannot determine whether the observed distributions are KD-specific or represent a further deviation from healthy values in KDSS. Our results showed that only CD4+ T cells were altered in the KDSS group, as has been reported in other KD studies. However, unlike previous studies, we observed a significant decrease in CD4+Vβ2+ T cells in patients with KDSS, suggesting activation and subsequent apoptosis, playing a role in the etiology of KDSS. Although we found no significant differences in the percentage of activation of CD4+Vβ2+ T cells between both groups of study, this effect can be attributed to activation-induced cellular death once lymphocytes are activated, which is consistent with the significant lymphopenia observed during flow cytometry analysis in samples from patients with KDSS. However, the study did not directly assess apoptosis, exhaustion or tissue homing. Redistribution to tissues and changes in peripheral-cell composition are alternative hypotheses that these measurements cannot distinguish. We also analyzed the presence of SAg-associated gene-positive bacterial isolates in patients’ stools. Cultures from stool samples and DNA were extracted to identify the presence of *sea*, *seb*, *sec*, *tst-1*, *speA*, *speB and speC* superantigens which were previously identified in patients with KD and coronary aneurysms. Our results only showed an association between *sea* and KDSS. These results allow us to consider that *sea* could activate CD4+Vβ2+ T cells and produce a severe inflammatory state causing the shock syndrome.

Regarding coronary aneurysms, the frequency of this cardiovascular manifestation in our patients (18%) was higher than that in the rest of the Latin American population (9.4%). The significant association of SAgs and the development of coronary aneurysms observed in this study is consistent with previous reports that show an elevated frequency of CD4+/Vβ2+ and CD8+/Vβ2+ T cells. This data supports the concept that the activation of infiltrating Vβ2+ T cells and the presence of staphylococcal exfoliative toxin are involved in the cardiovascular damage associated with KS [24,25,26]. In 2020, reports from Europe described a new entity associated with SARS-CoV2, characterized by fever, systemic hyperinflammation, multiorgan involvement with prominent GI symptoms and shock. This syndrome, referred to as COVID-19-associated multisystemic inflammation syndrome in children, presented with features of TSS and KD. In fact, at the present time, it is impossible to distinguish pre-pandemic KDSS from MIS-C clinically [28]. Interestingly, several studies have suggested that SARS-CoV2 may act as an SAg triggering the development of MIS-C [29,30]. In line with our results, Benezech et al. explored the peripheral-blood mononuclear cells in four children in whom Kawasaki disease shock syndrome had been diagnosed. The authors observed a Vβ21.3+ T-cell expansion in CD4+ and CD8+ T cells in one patient and CD8+ T cells in another; both patients also presented with an up-regulation of activation (CD38) and exhaustion (TIM3 and programmed death 1) markers within Vβ21.3+ CD4+ and CD8+ T cells, as has been reported in MIS-C. They concluded that MIS-C (and KDSS, or what the authors call Pre-COVID-19 SARS-CoV2 negative MIS-C) characterized by Vβ21.3+ T-cell expansion would appear to represent a severe pediatric condition that may be triggered by diverse pathogens [31]. Regarding the bacteria isolated from patients with KD and KDSS, no significant difference was observed between the two groups, but the high antimicrobial resistance is striking, which reinforces the hypothesis that the etiology of KD is of infectious origin and that high antibiotic resistance is a risk factor for the disease; however, further studies are needed to support this.

Important limitations of this study were observed. Only eight patients had KDSS, and the *sea* comparison included three positive observations overall. Estimates are consequently unstable and imprecise; the larger comparison group does not compensate for the small KDSS group. The single-center sample limits generalizability. Multiple exploratory comparisons also increase the possibility of chance findings. The *sea* result requires independent confirmation. Peripheral T-cell frequencies and CD25 expression do not establish the mechanism underlying the observed group differences.

## 5. Conclusions

In this small exploratory study, *sea* gene detection in cultured stool-derived bacterial isolates was more frequent in KDSS than in KD without shock. This finding was based on two *sea*-positive KDSS cases and was sensitive to exclusion of either case. *sea* may contribute to the pathogenesis of KDSS in a subset of patients with Kawasaki disease; however, this remains a hypothesis. The present data do not establish toxin production, systemic exposure or causality. But the presence of CD4+ T cells bearing TCRVβ2 reinforces the SAg hypothesis as an etiology of shock syndrome. In addition, each form of KD could have a different etiology, which could explain the inconsistencies in the detection of SAg in KD patients.

## Figures and Tables

**Table 1:** Sequence of primers purchased from Integrated DNA Technologies.

Gen	Sequence 5’–3′	Product Size (bp)	Ref. No.
*sea*	F: GAA AAA AGT CTG AAT TGC AGG GAA CA	561	49889774
	R: CAA ATA AAT CGT AAT TAA CCG AAG GTT C		49889775
*seb*	F: ATT CTA TTA AGG ACA CTA AGT TAG GGA	405	49889776
	R: ATC CCG TTT CAT AAG GCG AGT		49889777
*sec*	F: GTA AAG TTA CAG GTG GCA AAA CTT G	296	49889778
	R: CAT ATC ATA CCA AAA AGT ATT GCC GT		49889779
*speA*	F: CCA AGC CAA CTT CAG ATC	356	152994995
	R: CTT TAT TCT TAG GTA TGA AC		152994997
*speB*	F: CAA CCA GTT GTT AAA TCT CT	762	152994998
	R: CTA AGG TTT GAT GCC TAC AA		152994999
*speC*	F: TGT CTT ATG AGG CCT CTC	386	152995000
	R: ATC TGA TCT AGT CCC TTC		152995001
*tst-1*	F: TAC TAA TGA ATT TTT TTA TCG TAA GCC CTT	180	49889784
	R: TTC ACT ATT TGT AAA AGT GTC AGA CCC ACT		49889785

*sea*, staphylococcal enterotoxin A; *seb*, staphylococcal enterotoxin B; *sec*, staphylococcal enterotoxin C; *speA*, streptococcal pyrogenic exotoxin A; *speB*, streptococcal pyrogenic exotoxin B; *speC*, streptococcal pyrogenic exotoxin C; *tst-1*, toxic shock syndrome toxin-1; F: forward; R: reverse.

**Table 2:** Comparison of clinical manifestations at diagnosis between cases and controls.

Variables	KDSS Group (n = 8)	KD Group (n = 83)	*p* Value
Temperature (°C), median (P25, P75)	37 (37, 39)	37 (37, 38)	0.580
HR (respiration/min), median (P25, P75)	32 (28, 38)	26 (22, 32)	0.303
RR (heartbeat/min), median (P25, P75)	140 (134, 174)	121 (110, 149)	0.069
SBP (mm/Hg), median (P25, P75)	53 (46, 54)	60 (66, 65)	0.216
DBP (mm/Hg), median (P25, P75)	100 (79, 90)	97 (113, 100)	0.656
Form of KD (complete), n (%)	6/8 (75%)	57/83 (69%)	0.582
Arthralgia, n (%)	0/8 (0%)	6/83 (7%)	0.431
Fever, n (%)	2/8 (25%)	20/83 (31%)	0.294
BCGitis, n (%)	1/8 (13%)	20/83 (24%)	0.457
Non-purulent conjunctivitis, n (%)	8/8 (100%)	69/83 (83%)	0.207
Peeling hands and feet, n (%)	2/8 (25%)	9/33 (27%)	0.762
Diarrhea, n (%)	2/8 (67%)	21/83 (25%)	0.985
Abdominal pain, n (%)	4/8 (50%)	10/83 (12%)	0.005
Edema of the hands and feet, n (%)	5/8 (63%)	50/83 (60%)	0.901
Erythema of the mouth and pharynx, n (%)	7/8 (88%)	52/83 (63%)	0.160
Erythema of palms and soles, n (%)	5/8 (63%)	35/83 (42%)	0.269
Exanthema, n (%)	7/8 (88%)	63/83 (76%)	0.457
Irritability, n (%)	4/8 (50%)	46/83 (55%)	0.769
Red, dry, cracked lips, n (%)	7/8 (88%)	65/83 (78%)	0.542
Tongue in “strawberry”, n (%)	2/8 (25%)	46/83 (55%)	0.010
Vomit, n (%)	2/8 (25%)	29/83 (35%)	0.571
Evolution time (days), median (P25, P75)	7 (4, 12)	5 (4, 9)	0.972
Hospitalization (days), median (P25, P75)	7 (4, 10)	4 (3, 5)	0.292

SBP, systolic blood pressure; DBP, diastolic blood pressure; HR, heart rate; RR, respiratory rate, n, proportion of patients with specific condition.

**Table 3:** Comparison of superantigen frequency in patients diagnosed with Kawasaki disease shock syndrome (cases) and non-shock (controls).

Variable	KDSS Group n = 8 (10.1%)	KD Group n = 71 (89.8%)	*p* Value
SAg, n (%)	2/8 (25%)	26/71 (37%)	0.515
*sea*, n (%)	2/8 (25%)	1/71 (1%)	0.001
*seb*, n (%)	0/8 (0%)	3/71 (4%)	0.553
*sec*, n (%)	0/8 (0%)	4/71 (6%)	0.491
*tst*-1, n (%)	1/8 (13%)	16/71 (23%)	0.513
*speA*, n (%)	0/8 (0%)	2/71 (3%)	0.6306
*speB*, n (%)	0/8 (0%)	0/71 (0%)	-
spe*C*, n (%)	0/8 (0%)	2/71 (3%)	0.631

SAgs, superantigens; *sea*, staphylococcal enterotoxin A; *seb*, staphylococcal enterotoxin B; *sec*, staphylococcal enterotoxin C; *tst-1*, toxic shock syndrome toxin-1; *speA*, streptococcal pyrogenic exotoxin A; *speB*, streptococcal pyrogenic exotoxin B; *speC*, streptococcal pyrogenic exotoxin C. n (%), number and percentage of patients with the presence of a specific gen.

**Table 4:** Bacteria isolated from stools of patients with Kawasaki disease associated-shock syndrome (cases) and non-shocked Kawasaki disease (controls), prior to IVIG treatment.

Bacteria	KDSS Group	KD Group	Total	*p* Value
	(n = 8)	(n = 71)	(N = 80)	
*Enterobacter cloacae*, n (%)	5 (63%)	35 (50%)	40 (50%)	0.504
*Enterococcus faecium*, n (%)	0 (0%)	9 (12%)	9 (11.3%)	0.654
*Shigella* spp., n (%)	0 (00%)	7 (9%)	7 (8.8%)	0.449
*Escherichia coli*, n (%)	4 (50%)	17 (24%)	21 (26.3%)	0.054
*Staphylococcus haemolyticus*, n (%)	1 (13%)	13 (18%)	14 (17.5%)	0.954
*Staphylococcus sciuri*, n (%)	0 (0%)	9 (12%)	9 (11.3%)	0.376
*Staphylococcus aureus*, n (%)	4 (50%)	20 (29%)	24(30%)	0.622
*Streptococcus pyogenes*, n (%)	5 (63%)	20 (29%)	25 (31.3%)	0.206

n (%), number and percentage of positive cases.

## Data Availability

The data generated in the present study are included in the figures and tables of this article.

## Abbreviations

The following abbreviations are used in this manuscript:

KD	Kawasaki disease
KDSS	Kawasaki disease shock syndrome
SAg	superantigen
SEA	staphylococcus enterotoxin
SEB	staphylococcus enterotoxin B
SEC	staphylococcus enterotoxin C
TCR	T cell receptor
TSST-1	Toxic shock syndrome toxin-1
SPE	streptococcal pyrogenic exotoxin
IVGG	intravenous gamma globulin
MIS-C	multisystemic inflammatory syndrome associated to COVID-19
